# Biomonitoring and Subsequent Risk Assessment of Combined Exposure to Phthalates in Iranian Children and Adolescents

**DOI:** 10.3390/ijerph15112336

**Published:** 2018-10-23

**Authors:** Maryam Zare Jeddi, Mohamad Eshaghi Gorji, Ivonne M. C. M. Rietjens, Jochem Louisse, Yuri Bruinen de Bruin, Roman Liska

**Affiliations:** 1 Division of Toxicology, Wageningen University, Stippeneng 4, 6708 WE Wageningen, The Netherlands; maryam.zarejeddi@wur.nl (M.Z.J.); ivonne.rietjens@wur.nl (I.M.C.M.R.); jochem.louisse@wur.nl (J.L.); 2 Food Safety and Hygiene Department, School of Public Health, Tehran University of Medical Sciences, Tehran 14155-6559, Iran; m-gorji@alumnus.tums.ac.ir; 3 RIKILT, Wageningen University and Research, Akkermaalsbos 2, 6708 WB Wageningen, The Netherlands; 4 DG Joint Research Centre, European Commission, Directorate E—Space, Security and Migration, Knowledge for Security and Migration Unit (E.7), TP 450, Via E. Fermi 2749, 21027 Ispra (VA), Italy; 5 DG Joint Research Centre, European Commission, Directorate F—Health, Consumers and Reference Materials, Chemical Safety and Alternative Methods Unit (F.3), TP 126, Via E. Fermi 2749, 21027 Ispra (VA), Italy; roman.liska@ec.europa.eu

**Keywords:** anti-androgenic phthalates, human biomonitoring (HBM), daily intake (DI), mixture, maximum cumulative ratio (MCR)

## Abstract

This study aimed to estimate the exposure and related health risks of phthalates, and to assess the health risks from combined exposure to three of the phthalates sharing the same mode of action (anti-androgenicity) in children. We determined the internal exposure of 56 Iranian children and adolescents aged 6 to 18 years by analyzing seven urinary metabolites of five phthalates. The estimated daily intake values derived from the biomonitoring data ranged from 0.01 µg/kg bw/day for butyl benzyl phthalate (BBP), to 17.85 µg/kg bw/day for di(2-ethylhexyl) phthalate (DEHP). The risk assessment revealed that not only the exposure to the individual phthalates, but also the combined exposure to the three anti-androgenic phthalates (DEHP, DBP, BBP) did not raise a safety concern (hazard index values averaged 0.2). The range of maximum cumulative ratio values varied from around 1 for most individuals to around 2 in some individuals, indicating that the combined exposures were dominated by one and in some cases by two of the three anti-androgenic phthalates, especially dibutyl phthalate (DBP) and/or DEHP. Based on biomonitoring data, the overall combined exposure of Iranian children to phthalates does not raise a concern, while reduction of exposure is best focused on DEHP and DBP that showed the highest hazard quotient.

## 1. Introduction

Phthalates are diesters of phthalic acid with a wide variety of industrial applications. There is an interest in the safety evaluation of phthalate exposure because these compounds are ubiquitous environmental contaminants, resulting in widespread human exposure, with varied toxicological endpoints. Several phthalates have been cataloged as ubiquitous environmental endocrine-disrupting chemicals (EDCs) and that is why there is a strong demand for their reliable determination. Phthalates with endocrine-disrupting properties, such as di(2-ethylhexyl phthalate (DEHP), dibutyl phthalate (DBP) and butyl benzyl phthalate (BBP), are suspected to interfere with developmental androgen action, possibly leading to adverse effects on reproductive function [1,2]. Phthalates have been classified as “chemicals of concern” by the U.S. EPA [3]. In addition, the European Chemicals Agency’s Committee for Risk Assessment (RAC) recommends that the trends in exposure to phthalates (in consumer products, body burden based on biomonitoring, content in and migration from articles, etc.) should be monitored [4]. Certain phthalates, like DBP, BBP, DEHP, diethyl phthalate (DEP), and dimethyl phthalate (DMP) have numerous industrial applications and uses including food packaging, personal-care products, pharmaceuticals, medical devices, building materials, nutritional supplements, cleaning materials, solvents, adhesives, paints, lacquers, insecticides, children’s toys, and children’s school supplies which makes them omnipresent in the environment [5,6,7]. In these products phthalates are not chemically bound to the polymer matrices and therefore might easily migrate to the food and surrounding environment which leads to exposure of humans by multiple routes including mainly ingestion, inhalation and dermal uptake throughout their lifetime beginning in fetal stages [8,9,10].

Several animal studies reported that exposure to DBP, BBP, DEP, and DEHP has been associated with reproductive developmental damage, endocrine disruption and neurodevelopmental toxicity, and the European Food Safety Authority (EFSA) determined the critical effects of DEHP, BBP and DBP to relate to reproduction as derived from data from reproduction/developmental toxicity studies [11,12,13,14,15,16,17]. In addition, epidemiological studies, in spite of their limitations, suggest that there are strong and rather consistent indications that phthalates may affect reproductive outcome and children’s health [18]. Although phthalates have short biological half-lives, and are quickly excreted from the body as their respective metabolites (e.g., monoesters), particular consideration has been given to phthalates for years, especially due to their ubiquitous existence in the environment, the size of the population exposed and their endocrine-disrupting properties [18,19,20,21]. Phthalates can undergo metabolism in two stages in the human body namely phase I (hydrolysis, oxidation) catalyzed by esterases and lipases and phase II (conjugation) catalyzed by uridine 5′-diphosphoglucuronyl transferase enzyme. Lower molecular weight phthalates such as DEP, DMP and DBP will undergo phase I biotransformation to become hydrolytic monoester metabolites while higher molecular weight phthalates such as DEHP first will hydrolyze into a monoester and that can further be oxidized to secondary oxidized metabolites. Hydrolytic monoesters and secondary oxidized metabolites are further metabolized through phase II biotransformation to produce glucuronide conjugates. The metabolites are more soluble in water than the parent compounds and are excreted in urine [22,23,24].

Young children, as a result of their proportionally higher rates of breathing, eating and drinking, are likely to be more exposed per unit of body weight than adults [25]. A Canadian study reported children to display significantly higher urinary concentrations of metabolites of DEHP, DBP and BBP than adolescents and adults [26]. Similar results were also observed in German school children and the U.S. National Health and Nutrition Examination Survey (NHANES) and Demonstration of a study to Co-ordinate and Perform Human Biomonitoring on a European Scale’ (DEMOCOPHES) study [27,28], which is also in concordance with the results of other studies [29,30,31,32].

Human biomonitoring (HBM) is an important tool in the investigation of phthalate exposure and risk assessment since it reflects the phthalate body burden by measuring specific metabolites especially in the urine [33,34]. Although phthalate metabolites can be detected in several body fluids such as amniotic fluid, breast milk, saliva and seminal plasma [35,36,37,38,39], the presence of enzymes such as esterases in these matrices can cleave phthalates converting the phthalates samples from external sources into their monoesters [40]. In general, in epidemiological studies urine has been considered the matrix of choice for non-persistent chemicals, such as phthalates because urinary concentrations of especially their metabolites are usually considerably adequate biomarkers of exposure [21,41,42]. Therefore, the measurement of phthalate metabolites in urine as a valuable approach in environmental epidemiology studies, represents an integrated measure of exposure to phthalates from all possible known and unknown sources and routes, and incorporates individual variability in exposure profiles [21,43]. Forward and backward methods can be used for exposure assessment with the latter one being based on interpretation of HBM data [40]. Forward analysis uses measured intake doses to predict body burden while backward (reverse) analysis uses urinary HBM data to reconstruct past exposure by calculating estimated daily intake (EDI) [44]. One of the simplest methods to convert urinary HBM concentrations into exposure doses (e.g., EDIs) is based on the fractional urinary excretion (F_UE_), defining the fraction of the dose that ends up as a defined biomarker in a relevant matrix. Thus, the F_UE_ can be used for reverse dosimetry and convert the urinary level of a biomarker into an oral dose level. In this approach a correction for urinary dilution can be made applying the urine volume-adjustment method or the creatinine adjustment approach [40].

When considering the use of urinary biomarkers for estimation of phthalate exposure it is also of importance to consider combined exposure, since disregard of combination effects may lead to underestimation of risks [45]. For instance with a mixture of two phthalates, DBP and DEHP, which act through a common mode of action by suppressing testosterone synthesis, the combined effects were shown to be additive [46]. This illustrates the importance of cumulative risk assessment (CRA), considering the effects of combined exposure. In the case of phthalates, chronic co-exposure may constitute a risk of anti-androgenic effects during the stages of puberty due to hormonal changes and development of reproductive organs [47]. Therefore, the U.S. National Research Council in recent recommendations has reported that phthalates meet the conditions necessary to warrant a mixture risk approach [7].

The aim of the present study was to determine the extent of exposure to the phthalates BBP, DBP, DEHP, DEP and DMP, for the first time, among children and adolescents in Iran, and to estimate for this population the risk of exposure to the individual phthalates as well as to the combined exposure to the anti-androgenic phthalates BBP, DBP and DEHP.

## 2. Materials and Method

### 2.1. Study Population and Sample Collection

**Figure 1:**
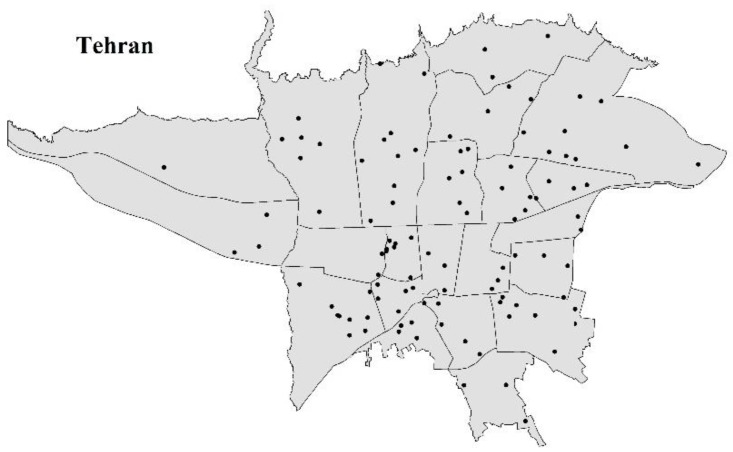
Location map of the study area showing the sampling sites.

This study is a cross-sectional study conducted among children and adolescents, ranging from 6 to 18 years of age in Tehran, Iran between September and November 2015. Fifty-six healthy children and adolescents were included in the study by random selection. Random house addresses for recruiting participants and collecting urinary samples were selected by a weighted approach based on the Tehran population density using ArcGIS software (ESRI, Redlands, CA, USA) (Figure 1).

From this initial list, house addresses for actual sampling were individually approached. For collection of the required spot urine samples, one resident per household was randomly chosen among the children and adolescents to participate in this study. If there was no child at the mentioned age range in the randomly selected house, the next selected house was interviewed to find an eligible case. Urine samples were collected in a sterile polypropylene cup. Samples were shipped on dry ice to the laboratory for the quantification of concentrations of phthalate metabolites. The subsets of the sample population were stratified by age (pre-reproductive: 6–11; reproductive: 12–18), and gender (girl and boy), as these factors have been indicated to have potential influence on phthalate exposure levels [48]. Demographic and anthropometric measures were assessed by trained health worker interviewers. Verbal and written assent was obtained from participants and their legal guardians. Further, this study was approved by the Research Ethics Committee of Tehran University of Medical Sciences (code No. 24039).

### 2.2. Target Compound and Analysis

Collected spot urine samples were analyzed for seven phthalate metabolites of five phthalates, namely, monobutyl phthalate (MBP) for DBP, monobenzyl phthalate (MBzP) for BBP, monoethyl phthalate (MEP) for DEP, monomethyl phthalate (MMP) for DMP, mono(2-ethylhexyl) phthalate (MEHP), mono(2-ethyl-5-hydroxyhexyl) phthalate (MEHHP), and mono(2-ethyl-5-oxohexyl) phthalate (MOEHP) for DEHP. All the investigated phthalate metabolites (99.9% purity) were purchased from Cambridge Isotope Laboratories, Inc. (Andover, MA, USA). Ammonium acetate (98%), 3-bromobenzoic acid (3-BrBA) (internal standard, I.S.) (99.9% purity), ferric chloride hexahydrate (FeCl_3_·6H_2_O), β-glucuronidase from *Helix pomatia* type H-2 and the derivatization reagents chlorotrimethylsilane (TMCS) and N,O-bis(trimethylsilyl)trifluoroacetamide (BSTFA) in pyridine (1:10:10) were purchased from Sigma-Aldrich (St. Louis, MO, USA). Multiwalled carbon nanotubes (MWCNTs, length 5.0–30 µm, diameter 30–60 nm), were obtained from Nanoshel (Panchkula, India) [49].

### 2.3. Phthalate Metabolite Analysis

The chemicals, reagents, applied methods for sample treatment and instrument analysis were reported in our previous study [49]. Briefly, two milliliters of human urine sample spiked with internal standard (10 µg/L) was buffered with 1.0 M ammonium acetate solution (100 µL, pH 6.8). To ensure complete deconjugation of phthalate metabolites, 20 µL β-glucuronidase from Helix pomatia type H-2 were added to the urine [50]. The sample was sealed in a glass tube at 37 °C and gently mixed for 90 min. The mixture was then acidified with phosphate buffer (1 M, pH 2, 1 mL). Then multiwalled carbon nanotube-magnetic nano particle (MWCNT-MNP) composite (100 µL of suspension 10 mg·mL^−1^) [49] and 100 mg NaCl were added and the mixture was vigorously vortexed for 3.0 min to extract the analytes. Then, an external magnet was applied to gather the magnetic adsorbent. After precipitation of the magnetic sorbent the supernatant was discarded followed by the addition of 5.0 mL isopropanol to elute phthalate metabolites from the adsorbent with vigorous vortexing for 2.0 min. The magnetic adsorbent was gathered using an external magnet afterward. The desorption solvent was collected and evaporated to dryness at 50 °C under a gentle stream of nitrogen. Then, 50 µL of derivatization reagent TMCS and BSTFA in pyridine (1:10:10) was added to the residue. The sample was mixed and kept at 65 °C for 30 min. Finally, 1 µL of the resulting solution was injected into the GC-MS that was an Agilent gas chromatograph 6890 plus (Agilent Technologies, Palo Alto, CA, USA) equipped with a 5973 quadrupole mass spectrometer. One blank, one repeat and one quality control (QC) recovery sample of high and low concentration were included in each analytical run to monitor for accuracy and precision. Blank samples, contained 3-bromobenzoic acid (3-BrBA) as internal standard (I.S.) and the concentration of blank samples was required to be less than twice the method detection limit [49]. Blank samples and QC samples were analyzed at the beginning, middle and at the end of the sample queue [51]. The limit of detection (LOD), limit of quantification (LOQ), linearity, accuracy, intra-day and inter-day precision and specificity of the method applied were and reported in our previous work [49]. The LODs varied for each phthalate metabolites and ranged from 0.025 to 0.050 µg/L (Table 1) and the LOQs were between 0.125–0.250 µg/L.

In addition, a satisfying reproducibility was reported by evaluating the intra-day precision and inter-day precision with relative standard deviations (RSDs) less than 11.2% and 11.4%, respectively. The recoveries of the phthalate metabolites ranged from 92.6% to 98.8% with the RSDs less than 10.7% [49].

Creatinine adjustment was used to correct for urine dilution. Urine creatinine concentrations were determined according to the Jaffé method. It has been reported that some β-glucuronidases from *Helix pomatia* (also used in the present study) may contain environmental phthalate contaminants that may lead to phthalate metabolite measurement errors in urine [56,57]. However, the results of the blank samples of the present study and from another study [50] revealed that using the abovementioned enzyme did not influence the analytical results. Furthermore β-glucuronidases from *Helix pomatia* may contain some lipase/arylsulfatase activity which hydrolyse fatty acid and sulfate ester bonds [58]. However, in the present study the major phthalate conjugates were expected to be glucuronide conjugates. Lipid esters were not expected and sulfate esters only to a limited extent while upon their hydrolysis by the arylsulfatase present in the enzyme preparation they have been included in the analysis.

### 2.4. Daily Intake Estimation

Based on the spot urine phthalate metabolite concentrations (expressed in µg/L urine), the estimated daily intake (EDI) in μg/kg body weight/day of the main phthalates was determined. The EDIs of the target phthalates (DEP, DEHP, DBP, DMP, and BBP) were calculated for each child normalized based on urine creatinine extraction. The EDI was estimated using the following Equation (1) [43,48]:

(1) $$\mathrm{EDI}=\frac{{\mathrm{UC}}_{Cr-adj}\times \mathrm{CE}}{F_{\mathrm{UE}}\times 1000_{mg/g}}\times \frac{{\mathrm{MW}}_{Parent}}{{\mathrm{MW}}_{Metabolite}}$$

where EDI is the estimated daily intake of phthalate (μg/kg body weight/day), UC*_Cr-adj_* is the phthalate metabolite concentration adjusted for creatinine ((μg/mL urine)/(g creatinine/mL urine)), CE is the daily (24 h) creatinine excretion normalized to body weight (mg/kg body weight/day); and F_UE_ is the fractional urinary excretion of the metabolite determined over the full-time course of urinary excretion (24 h excretion) in relation to ingested parent phthalate over 24 h after exposure (‘unitless’); 1000 is the unit conversion factor creatinine (mg/g); MW*_parent_* and MW*_metabolite_* are the molecular weights of parent phthalate and the metabolite (mg/mole) of each phthalate, respectively. Values related to F_UE_, MW*_parent_* and MW*_metabolite_* are displayed in the Table 1. The CE was calculated based on the equations from [59] for children and adolescents (aged 6–18 years) by using demographic information (age, gender, height, and weight) [59].

We calculated DEHP exposures based on each individual metabolite and in addition based on the sum of the three measured urinary DEHP metabolites, MEHP, MEHHP and MEOHP in µg/g creatinine. Since the calculation of the EDI for DEHP is based on multiple metabolites, the following Equation was used (2) [48]:

(2) $$\overset{j}{\underset{i=1}{\sum }}\mathrm{EDI}={\mathrm{EDI}}_{i}\times \frac{F_{\mathrm{UE}i}}{\sum_{i=1}^{j}F_{\mathrm{UE}i}}$$

where *i* is the *i*th metabolite of a phthalate, *j* is the number of metabolites for a phthalate, and F_UEi_ is the fractional urinary excretion of the *i*th measured DEHP metabolite and the amount of DEHP taken up (‘unitless’).

In addition, the relative metabolic rate (RMR) of DEHP metabolism was calculated to investigate the possible differentiations in DEHP metabolism among population groups. The formation of the three DEHP metabolites analyzed in this study occurs in a stepwise metabolic pathway [60] including DEHP conversion to MEHP (1st step), MEHP hydroxylation to MEHHP (2nd step) followed by MEHHP oxidation to MEOHP (3rd step). Based on quantification of the 3 DHEP metabolites two relative metabolic rate (RMR) values can be determined. The first relative metabolic rate (RMR1) is representative for the rate of MEHP hydroxylation to MEHHP and calculated by dividing a molar concentration (µmol/μL) of MEHHP as a product over that of MEHP as a precursor. Similarly, the second relative metabolic rate (RMR2) is representative for the rate of MEHHP oxidation to MEOHP and calculated by dividing a molar concentration (µmol/μL) of MEOHP over that of MEHHP [61].

### 2.5. Mixture Risk Assessment

Hazard quotients (HQs) were calculated for quantifying potential risks for children and adolescents from exposure to single phthalates, which is defined as the ratio between the EDI and their health-based guidance values and/or acceptable level of exposure listed in Table 2 and comprising the Tolerable Daily Intake (TDI), the Reference Dose (RfD)and the Reference Dose for Anti-Androgenicity (RfD-AA) established by EFSA, U.S. EPA and Kortenkamp and Faust (2010) [62], respectively. The value of HQ is described in Equation (3) for participant *i* and phthalate *j*:

(3) $${\mathrm{HQ}}_{ij}=\frac{{\mathrm{EDI}}_{ij}}{{\mathrm{AL}}_{ij}}$$

The health-based guidance values and/or acceptable level of exposure is denoted AL in Equations (3) and (4).

To assess combined exposures to multiple phthalates, the Hazard Index (HI) was used, which is based on the dose addition concept [7,69,70]. The following Equation was applied to determine the values HI for participant *i* and phthalate *j* for N phthalates (4):

(4) $$\mathrm{HI}=\overset{n}{\underset{i=1}{\sum }}\frac{\mathrm{EDI}}{\mathrm{AL}}$$

where EDI is the estimated daily intake of phthalate (μg/kg body weight/day), and *n* is the number of substances in the mixture. The selected health-based guidance values and/or acceptable level of exposure used to construct the HQ and HI on the basis of the same toxicological endpoint (anti-androgenic effects) are two different sets which include: (1) The tolerable daily intake (TDI) values as derived by the European Food Safety Authority (EFSA) for DBP, BBP, and DEHP based on testicular/germ cell toxicity [63,64,65]. The reference doses for anti-androgenicity (RfD-AA) were established by Kortenkamp and Faust for DBP, BBP, and DEHP [62].

The United States Environmental Protection Agency Reference Doses (U.S. EPA RfDs) were not used, because the RfDs for DBP, DEHP and BBP have not been derived based on anti-androgenic effects as the most sensitive endpoint [66,67,68]. The EFSA TDIs, the RfD-AA values and the EPA RfD values for DBP, DEHP and BBP are presented in Table 2. Thus, two different HIs were estimated based on two types of health-based guidance values. DEP and DMP were not included in the combined risk assessment because these two phthalates do not exhibit endocrine disrupting effects similar to those observed for DBP, BBP and DEHP. Indeed, predominantly negative results on the oestrogenic or antiandrogenic effects of DEP and DMP have been reported and no endocrine-related adverse effects of DEP and DMP on the male reproductive system have been observed and these phthalates are regarded safe compared to other phthalates [7,71,72]. Nevertheless, they may contribute significantly to other adverse effects for example neurodevelopmental disorders [73].

The present study also applied the Maximum Cumulative Ratio (MCR) approach, which is the ratio of the combined risk measure HI divided by the HQ of the chemical with the highest risk contribution (HQ*_max_*) [74]. Indeed, this tool can be used to determine which chemicals are the drivers of the combined risk [73]. This approach helps to prioritize chemicals for mitigation strategies as well.

The maximum HQ among the investigated phthalates in the current study was determined taking into account three phthalates (*j* = 3), and the maximum HQ (HQ*_M_*) was determined as indicated in Equation (5):

HQ*_M,I_* = *Max* HQ_*ij*_ (5)

The value of MCR for a participant *i* in an exposed population is defined as [75]:

(6) $${\mathrm{MCR}}_{i}=\frac{\mathrm{HI}}{{\mathrm{HQ}}_{M,i}}$$

The values of MCR will vary across participants in an exposed population ranging from 1 to *N* (i.e., MCR*_i_* ∈ [1, *N*]), where *N* is the number of chemicals considered in the assessment. When ratio values for a mixture are close to 1, one chemical is responsible for nearly all of the individual’s combined risk. A value of *N* indicates that, the individual receives an equitoxic dose from all chemicals [75].

### 2.6. Statistical Analysis

Appropriate descriptive statistics were used for description of all metabolite concentrations, demographic variables, RMR, HQs, HIs and MCRs. Nonparametric Mann-Whitney U test was implemented to compare median of target variables across sex and age groups. Principal component analysis (PCA) was applied to investigate the potential exposure sources and Spearman’s rank correlation coefficient were used for investigation correlations between metabolites. Data were analyzed using STATA Version 12. (StataCorp, College Station, TX, USA). Statistical significance was set at *p* < 0.05. Concentrations of phthalate metabolites below LOQ were set equal to a value of ½ LOQ, and concentrations <LOD were set to 0.

## 3. Results

### 3.1. Urinary Phthalate Metabolite Concentrations

This study is based on data from 56 participants (24 males and 32 females) aged 6 to 18 years. All phthalate metabolites were detected in concentrations above the respective LODs, which indicated the ubiquitous exposure of Iranian children and adolescents to phthalates. MzBP as a metabolite of BBP was detected and quantified in 92.9% (52 out of 56) of the urine samples and was present in 4 samples below the LOQ for MzBP of 0.25 μg/L, so the amounts present in these 4 samples could not be quantified. Descriptive statistics and statistical comparisons across gender and age groups of seven phthalate metabolites are presented in Table 3. The median unadjusted and creatinine-adjusted levels of MBP, MEHP, MEHHP, MEHOP, MMP in girls aged 6 to 18 years (*n* = 32) were slightly lower than those in boys aged 6 to 18 years (*n* = 24). For MEP and MBzP, the highest concentrations were measured in girls with statistically significant differences compared to values for boys. Median levels of phthalate metabolites appeared not considerably different between the age groups and differences were indeed shown to be not statistically significant. This trend was consistent also for the creatinine-adjusted values (Table 3). In this study, the geometric mean (GM) concentration of the sum of DEHP metabolites (Σ3DEHP metabolites) was 47.7 µg/L with a decreasing order in the level of the three DEHP metabolites that varied as follows: MEHHP > MEOHP > MEHP. In addition, MBP and MEP were the most abundant metabolites, with GM values of 36.4 and 27.5 µg/L respectively. MBzP showed the lowest level in both unadjusted and creatinine adjusted models with a GM value equal to 2.0 µg/L. Table 4 depicts Spearman correlation coefficients among several selected urinary phthalate metabolites. As shown, statistically significant Spearman correlations (*p* < 0.05) were identified between some of the metabolites. Our study displayed a strong correlation between the primary metabolite of DEHP, MEHP, and the two oxidative metabolites of DEHP including MEHOP and MEHHP as well as between these two secondary metabolites of DEHP. A moderate correlation was indicated between MBP and MEP or MMP, and between MBzP and MEP or MBP. The observed correlations were correlated positively (*p*-value < 0.05; Table 4), an observation that may indicate combined exposure.

**Figure 2:**
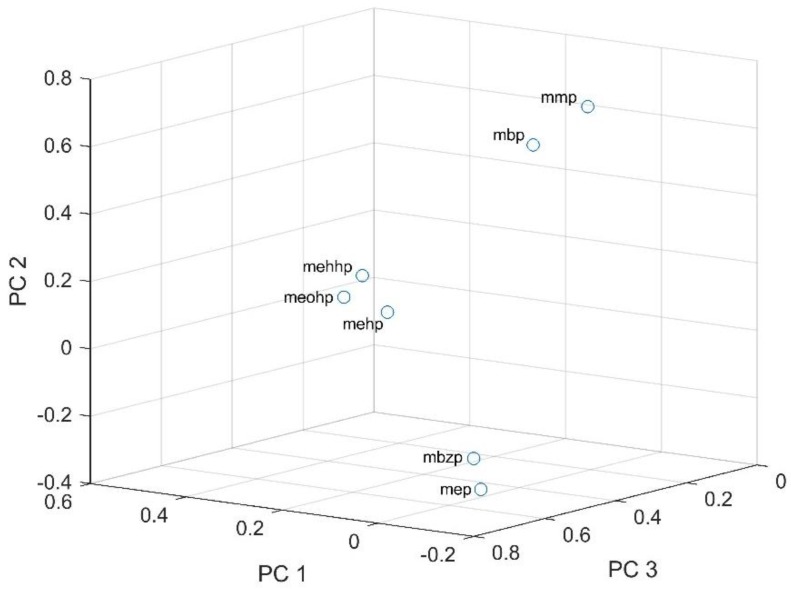
Principal component analysis (PCA) of the levels of the seven phthalate metabolites detected in urine samples from Iranian children and adolescents.

In addition, PCA was performed for the seven phthalate metabolites (MMP, MEP, MBP, MBzP and the three DEHP metabolites). The results of PCA are summarized in Figure 2. Three significant (Eigen values > 1.000) factors were retained and they can explain about 83% of the data variability. As shown in Figure 2, seven investigated phthalates are in three clouds. The first cloud is represented by MBzP and MEP, the second by the three DEHP metabolites (MEHHP, MEOHP and MEHP) and the third one by MMP and MBP. This clustering in the PCA analysis might point at a similar molecular origin, combined exposure, and or/similar ADME (absorption, distribution, metabolism and excretion) characteristics.

In this study, we included all samples in the relative metabolic rate (RMR1 and RMR2) calculation because all three metabolites of DEHP were detected in 100% of the samples. The RMR1 and RMR2 arithmetic mean for children and adolescents were 13.4 and 0.72, respectively. Mean of RMR1 in girls (13.6) was slightly (but not statistically significant) higher than that in boys (13.4) and also no gender- or age -based differences were observed for either RMR1 or for RMR2.

### 3.2. Daily Intake Estimations and Combined Risk Assessment

The estimation of individual daily phthalate intakes among the study population was performed according to the creatinine adjusted model. The median EDIs of BBP, DMP, DEP, DBP, and DEHP for all investigated children and adolescents were 0.06, 0.8, 1.0, 1.1, and 3.4 μg/kg body weight/day, respectively (Table 5). Comparing boys and girls in the whole age range, the EDIs of DBP, DEP and BBP for girls were slightly higher than those for boys with statically significant differences for DEP. Concerning age, no statically significant differences were found among daily intake values for all investigated phthalates.

On the basis of the EDI values for each participant, the risks (HQs as well as HI) associated with phthalate exposure based on EFSA TDI values and RfD-AA values were characterized. Results obtained are summarized in Table 6. In this study, HQ reflects the risk value for a single phthalate, while HI shows the risk value obtained for cumulative exposure of the anti-androgenic phthalates BBP, DBP and DEHP. As shown in Table 6, median HIs for cumulative exposure based on both health-based guidance values (EFSA TDI and RfD-AA) are below 1. HIs ranged from 0.03 to 0.70 and from 0.02 to 0.62 based on EFSA TDI and RfD-AA approaches, respectively, and the HIs did not exceed 1 for any of the surveyed participants.

The MCR calculated among the 56 participants ranged from 1.09 to 2.32. Because in the present study, three phthalates were considered, MCR values can theoretically range between 1 and 3. An MCR value close to 1 indicates that one chemical had a dominant influence on the participant’s value of HI. The fact that all MCR values are between 1.1 and 2.32 indicates that for none of the exposed participants the three phthalates had the same influence on the participant’s value of HI, since this would have resulted in an MCR value of 3.

That is, for each subject, a subgroup of phthalates had a dominant influence on the participant’s value of HI. The collective internal doses of all participants were driven by either DBP or DEHP. Among investigated phthalates, BBP did not produce HQ_M_ for any participants. Approximately, 73% of combined HI-TDI could be attributed to DBP’s metabolite MBP while based on the HI-RfD-AA the sum of DEHP metabolites makes up the whole RfD-AA.

## 4. Discussion

In this study, we used a human biomonitoring approach to determine EDI values for five different phthalates in Iranian children and adolescents, based on phthalate metabolite levels in urine spot samples, and performed an associated risk assessment. For this risk assessment the EDI values obtained were compared to two sets of health-based guidance values as acceptable levels of exposure, derived based on anti-androgenic effects as the critical endpoint, including TDI values established by EFSA [63,64,65] and RfD-AA values determined by Kortenkamp and Faust [62] using the HQ approach. The evaluation also included a combined risk assessment for exposure to phthalate mixtures using the HI approach.

**Figure 3:**
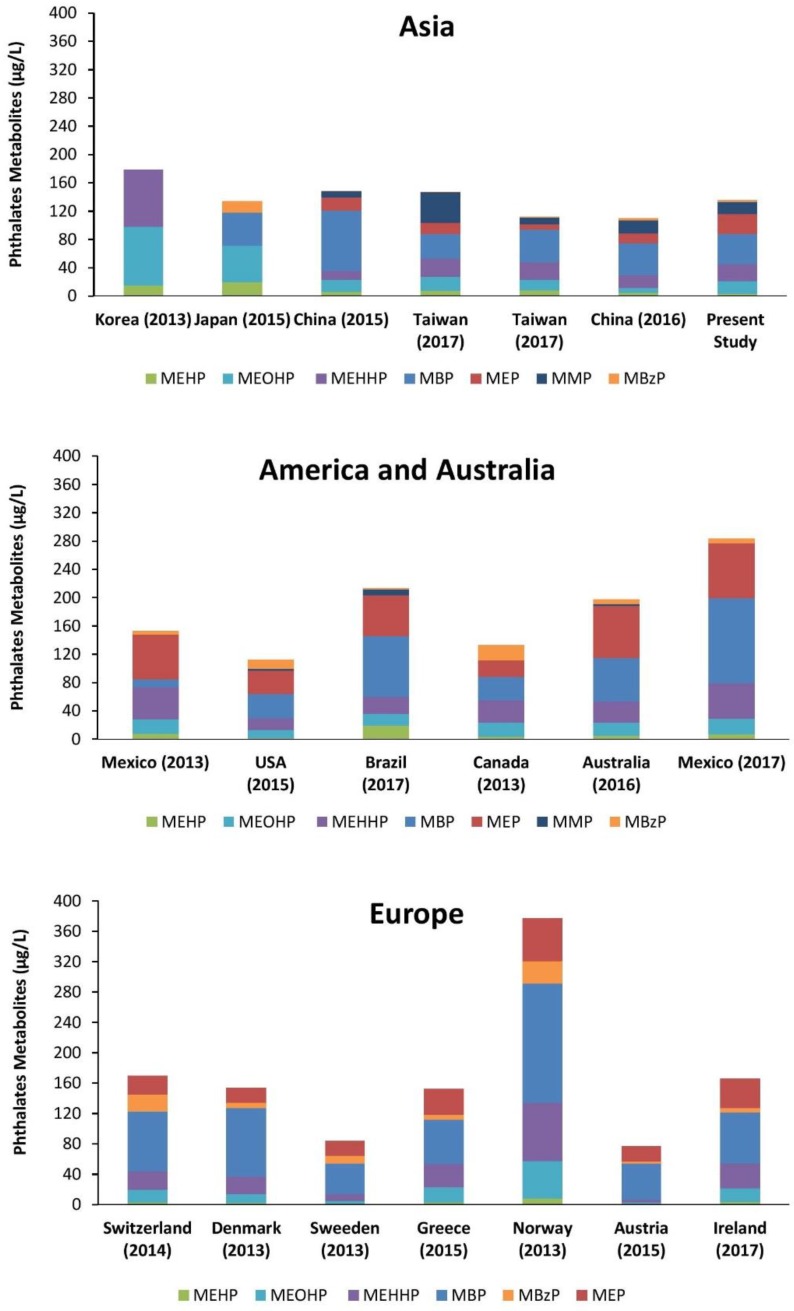
Median urinary concentrations (µg/L) of phthalate metabolites throughout the world. Related references based on regions are as follows: Asia: [61,87,88,89,90,91], America and Australia: [23,76,77,78,79], Europe: [32,47,92,93,94]. All the reviewed studies were conducted among children and adolescents (<18 years old) with sampling time after 2010.

The results of this study show that Iranian children and adolescents are ubiquitously exposed to certain phthalates included in the study, namely DEHP, DBP, DEP and DMP. Our findings showed that the levels of the urinary biomarkers for phthalate exposure varied in the order MBP > MEP > DEHP metabolites > MMP > MBzP. For comparison the results from other studies on exposure of children and adolescents to phthalates as reported in the literature on a worldwide scale are presented in Figure 3. The urinary metabolite patterns obtained in our study are especially similar to the urinary phthalate metabolite patterns reported for children and adolescents from other countries such as Taiwan, China, Brazil and Greece [76,77,78]. All the reviewed studies were population-based cross-sectional studies conducted among minors (<18 years old) after 2010. Consistent with our results, among all studies the lowest urinary concentrations were observed for MBzP. This reveals that children and adolescents have relatively lower exposure to BBP compared to other phthalates, and that this is the case in several countries over the world, probably reflecting that the application and sources of exposure to BBP are comparable [4]. The urinary metabolite patterns in our study revealed also differences to urinary metabolite patterns reported in several of the other countries for which data were available (Figure 3). Such differences may reflect differences in patterns of exposure in different countries due to country specific use patterns for phthalates in relevant products, differences in food consumption habits, and/or differences in socio-economic strata [79]. These differences may also reflect changes in the phthalate content of specific products over time, and further may be the result of public pressure and political regulations [21]. For example in 2004, the European Union (EU) has banned the use of certain phthalates including DEHP, DBP and BBP from cosmetics and food packaging (Directive 2004/93/EC) and in 2005 from all toys and childcare products (Directive 2005/84/EC) [80]. Furthermore, comparison of data from two studies in Denmark with sampling in the period 2006 to 2008 or more recent in 2011 revealed a decreasing trend in phthalate exposure [32,81]. Reported urinary concentrations for BBP, DBP and DEHP in the studies conducted by [32,81] (sampling time between 2006–2008 on 517 boys aged 6 to 19 years) are 1.5, 6.8 and 2 times higher than the results of the study in 2011 on 143 participants aged group of 6 to 11. Likewise, in another study with the same sampling year conducted by Frederiksen et al. on 725 Danish girls (ranged from 5.6 to 19.1 years) the level of phthalates are higher than the latest study carried out by Frederiksen et al. in 2013 [32]. This decreasing trend in phthalate exposure also becomes apparent when comparing two studies in the USA one with 2005–2006 and one with 2009–2010 as sampling periods [82]. In this comparison, the study populations were comparable with regard to age group, sex, sample size and country. This reduction in phthalate exposure is likely attributable to prohibition of usage of specific phthalates. In addition, dietary habits and life styles play an important role in exposure to phthalates [83]. One remarkable difference in the urinary phthalate metabolite pattern between the Iranian children and adolescents and data from several other countries was the fact that the Iranian samples revealed the presence of MMP, the metabolite of DMP. This metabolite was also observed to a significant extent in 6 to 18 year old children in China and Taiwan [77,84]. The possible sources of this DMP exposure are not clearly known. A study conducted in China suggested that the concentrations of DMP in milk products, instant noodle, cakes, cookies and salt eggs were higher than those in other foods [85]. DMP was detected in some food samples such as yogurt, fish, and spice from Europe and North America as well [86].

According to the results of the current study and in line with results from related studies, the concentrations of the oxidative metabolites of DEHP (MEHHP and MEOHP) appeared to be excreted in several-fold higher concentrations than MEHP the metabolite resulting from hydroxylation of DEHP (Figure 3). Although metabolic abilities may differentiate between age groups, previous studies already showed that these oxidative metabolites could be more sensitive biomarkers for monitoring exposure to DEHP than MEHP [95]. In addition, previous studies reported that children had a particularly faster relative metabolic rate (RMR) than adults, specifically for the first step of DEHP metabolism (RMR1: ratio MEHP/MEHHP) [61]. The results from the present study also corroborate that RMR1 is higher than RMR2, because the transformation of MEHP to MEHHP (as expressed by RMR1) appears to be positively related with age implying a reduced ability of DEHP metabolism at lower ages [94,95].

Furthermore, the urinary biomarker patterns confirmed the observation that Iranian children and adolescents, like children and adolescents in other countries, are simultaneously exposed to mixtures of phthalates. The PCA analysis indicated grouping of some phthalates (DEHP, DBP, DMP) and thus pointed at combined exposure from similar sources, like food packaging material and several consumer products, leading to combined exposure to DBP, DMP, and DEHP, and or/similar ADME [86]. In contrast, MEP and MBzP were positively correlated with PC3, which could be an indication of the same origin of exposure via for example personal care-hygienic products and cosmetics [96] which may explain why the concentrations of these two phthalates were higher in girls with statistically significant differences compared to values for boys. Generally, multiple phthalates correlate with one another if they are used in the same applications and thus share similar sources of exposure, and again there may be some other existing unknown sources of exposure [96].

In a review conducted by Smith et al., aiming to prioritize hazardous chemicals in children’s products based on the U.S. Children’s Safe Product Act (CSPA) database, the relationships between phthalate exposure and adverse health effects was investigated [96]. Four endpoints including endocrine disruption, reproductive and developmental toxicity, carcinogenicity and neurotoxicity were selected as relevant health endpoints in their framework. The analysis confirmed that toxicity drives a substantial part of the differences in health effects caused by the chemicals. Phthalates, including DEHP, BBP, and DBP were found to raise a concern because of reproductive and developmental toxicity, which would be in line with their activity as anti-androgens [96].

Across the whole study population of the present study, the highest median phthalates intakes were for DEHP within the range of 0.58 to 17.85 µg/kg body weight/day and DBP within the range of 0.2 to 3.1 µg/kg body weight/day. Median BBP daily intakes were lowest, ranging from 0.01 to 0.23 µg/kg body weight/day.

The phthalate exposure profile was consistent with previous studies. These studies also revealed that levels of most urinary phthalate metabolites detected for children were found to decrease with increasing age [97,98,99]. However, no significant differences were found between the two age groups in our study.

According to the results of our study, DEHP is the compound with the highest median HQ of 0.14 when based on the RfD-AA for anti-androgenic effects, whereas based on the TDI approach DBP with a median of 0.11 is the compound with the highest HQ value. However, since all HQ values, and also the HI values for combined exposure were markedly below 1.0 it can be concluded that for none of the surveyed participants the HQ and HI values raised a concern. It is also of interest to note that for the risk assessment performed, HQs and HIs were not calculated based on U.S. EPA RfDs, since these health based-guidance values are not based on endpoints that share an underlying mode of action. However, even when using U.S. EPA RfD values, EDI values for investigated phthalates in the present study would remain far below these health-based guidance values and thus corroborate that the exposure does not raise a concern. This result is in contrast to those from a study by Wittassek et al. (2007) conducted among German children (*n* = 239, 2–14 years old), in which some individuals showed DEHP exposure estimates based on HBM data that exceeded the U.S. EPA RfD for DEHP (20 μg/kg body weight/day) [100]. More so, Kim et al. (2014) in Seoul, using the U.S. EPA RfD value, approximately 3–8% of elementary school children (*n* = 39, aged 9–12 years) showed a HQ greater than 1.0 for DEHP exposure, using HBM data for exposure estimation [101]. The HI approach used in the present study for combined risk assessment has been previously used for combined risk assessments on phthalates in the literature. In a study conducted by Søeborg et al. (2012) [102] 129 Danish children and adolescents, 19 children exceeded the HI value of 1.0 determined based on EFSA TDI values for the anti-androgenic phthalates (DBP, BBP and DEHP), while one child exceeded the HI value of 1.0 based on the RfD-AA values [102]. Dewalque et al. (2014) also reported HI values based on TDIs exceeding the value of 1.0 in 25% of the children in a study on phthalate exposure of 52 male and female children (1–12 years) in Belgium [103]. A study conducted among Austrian children aged 7–15 years on cumulative risk assessment for combined phthalate exposure demonstrated that in 4.2% of children the HI values calculated based on TDIs were more than 1.0 [47]. In the present study, the HQ values for DBP based on EFSA-TDI values were higher compared to the estimated HQs based on RfD-AA values. This discrepancy is due to the fact that the underlying RfD-AA value used for DBP by [62] is 10 times higher than the relevant EFSA-TDI value (Table 2). However, DEHP was associated with the highest HQ value (~0.6) for Iranian children in RfD-AA approache. Thus, obviously, the HQ and HI values and resulting conclusions may to some extend depend on health-based guidance values used to calculate these values.

Regarding the MCR approach, the cumulative exposures of concern mainly originated from one of the three anti-androgenic phthalates including DBP and DEHP. The MCR approach has been applied to biomonitoring data on mixtures of dioxin-like chemicals [73], exposures to mixtures of chemicals in water [74,104,105], and mixtures in residential indoor air [106]. A recent study conducted by [75] was the first publication that used the MCR approach in a biomonitoring study on phthalates collecting data on six phthalates. The results of that study showed that HI values in the surveyed participants averaged 0.15 (HI < 1.0). Only 21 (0.8%) of the participants had HI values >1.0 [75]. Reyes and Price, reported that for about 43% of these participants with HI >1.0, a potential risk would have been overlooked if only single chemical based risk assessment (HQ) rather than a combined exposure approach (HI) was performed. In addition, the MCR calculated among the participants ranged from 1.1 to 3.6, which indicated that a single or a subgroup of phthalates like DEHP and DBP had a dominant influence on the participant’s value of HI [75]. Also in the present study, the HI values were dominated by specific phthalates, being DBP when determining HI values based on TDI values, and DEHP when determining HI values based on RfD-AA values.

Altogether, it can be concluded that, in line with other studies, our subjects were not exposed to single phthalates, but rather to a mixture of phthalates. In a previous study prioritizing chemicals and products, DBP, BBP and DEHP were identified as the highest priority chemicals based on both exposure and toxicity scores [96]. Metabolites of these priority phthalates were also detected in the urinary samples of the present study and were shown to contribute to the combined HI values. This corroborates that biomonitoring data indicate that the overall combined exposure to phthalates of Iranian children does not raise a concern, while reduction of exposure is best focused on DEHP and DBP that showed the highest HQ.

## 5. Conclusions

This work investigated human biomonitoring derived phthalate exposure data for a population of Iranian children and adolescents ranging from 6 to 18 years of age analyzing urinary biomarkers for five phthalates. The data indicated that the Iranian children and adolescents were exposed to a mixture of phthalates and a subsequent risk assessment revealed that none of the surveyed participants had HQ and HI values that raised a concern. The phthalates exposure pattern for the study population of Iranian children and adolescents appeared comparable to the results reported for children from other parts of the world with the greatest similarity being found for children from China, Taiwan, Brazil and Greece. Although combined exposure to anti-androgenic phthalates did not exceed the acceptable level of exposure, aside the investigations of phthalates in this study, people typically come into contact with several chemicals with anti-androgenic properties, which may also contribute to combined anti-androgenic effects. This indicates that a risk assessment of combined exposure including other anti-androgenic chemicals would be required to determine whether combined exposure to anti-androgenic chemicals is below acceptable levels.

## Figures and Tables

**Table 1:** Molecular weights and urinary excretion fractions for phthalate metabolites.

Parent Phthalate Compound	Phthalate Monoester Metabolite	Limit of Detection (LOD) (µg/L)	Molecular Weight of Diester Parent Compound, g/mole (MW*_parent_*)	Molecular Weight of Metabolite, g/mole (MW*_metabolite_*)	Urinary Excretion Fraction (FUE, 24-h), Expressed as Percent (%) *
Di-2-ethylhexyl phthalate (DEHP)	Mono-(2-ethylhexyl) phthalate (MEHP)	0.050	390.56	278.34	6.2
	Mono-(2-ethyl-5-hydroxyhexyl) phthalate (MEHHP)	0.050	390.56	294.35	14.9
	Mono-(2-ethyl-5-oxohexyl) phthalate (MEOHP)	0.050	390.56	292.33	10.9
Dibutyl phthalate (DBP)	Monobutyl phthalate (MBP)	0.040	278.34	222.24	84
Butylbenzyl phthalate (BBP)	Mono-benzyl phthalate (MBzP)	0.050	312.36	256.25	73
Diethyl phthalate (DEP)	Monoethyl phthalate (MEP)	0.025	222	194	69
Dimethyl phthalate (DMP)	Monomethyl phthalate (MMP)	0.030	194	180	69

* The F_UEs_ values are taken from the following studies: DEHP [52]; DBP [53]; BBP [54]; DEP, DMP [55].

**Table 2:** Related health-based guidance values and toxicological target for dibutyl phthalate (DBP), butyl benzyl phthalate (BBP) and diethylhexyl phthalate (DEHP).

Phthalate	Toxicological Target			Toxicity Health-Based Guidance Values		
	EFSA TDI	U.S. EPA RfD	RfD-AA	EFSA TDI (µg/kg bw/day) ^1^	U.S. EPA RfD (µg/kg bw/day) ^2^	RfD-AA (µg/kg bw/day) ^3^
**DBP**	Germ cell development and mammary gland changes	Increased mortality	Suppression of testicular testosterone production	10	100	100
**BBP**	Anogenital distance change	Increased liver-to-body weight and liver-to-brain weight ratios	Suppression of testicular testosterone production	500	200	330
**DEHP**	Testicular toxicity and developmental toxicity	Increased relative liver weight	Nipple retention	50	20	30

^1^ EFSA 2015 [63,64,65]; ^2^ IRIS EPA [66,67,68]; ^3^ Kortenkamp and Faust [62].

**Table 3:** Urinary concentrations of phthalate metabolites (in µg/L (µg/g creatinine)) in Iranian children and adolescents population (*n* = 56).

Phthalate Metabolites	Group	*n*	GM		5th		50th		95th		Max		*p*-Value ^a^	
**MEHP**	**All**	56	2.81	(2.82)	0.48	(0.53)	3.3	(3.3)	9.1	(11.5)	11.6 (13.2)		0.406	(0.579)
	≥6~<12	22	2.9	(3.02)	0.54	(0.55)	4.0	(3.6)	7.83	(8.72)	9.1 (11.5)			
	≥12~≤18	34	2.8	(2.7)	0.485	(0.53)	3.2	(3.3)	9.3	(12.1)	11.6 (13.2)			
	Boys	24	3.70	(3.61)	1.0	(0.65)	4.9	(4.1)	9.1	(12.1)	11.6 (13.2)		0.213	(0.127)
	Girls	32	2.35	(2.33)	0.32	(0.32)	2.8	(2.6)	7.83	(7.2)	9.3 (9.5)			
**MEOHP**	**All**	56	18.14	(18.12)	3.62	(3.6)	17.5	(19.3)	65.1	(73.8)	79.43	(99.4)	0.470	(0.233)
	≥6~<12	22	19.5	(20.3)	4.0	(3.7)	21.43	(24.2)	58.3	(46.2)	63.7	(121.1)		
	≥12~≤18	34	17.3	(16.9)	3.6	(2.2)	16.62	(18.0)	69.33	(82.7)	79.43	(99.4)		
	Boys	24	19.32	(19.1)	4.0	(3.7)	19.9	(20.4)	60.64	(82.74)	79.43	(99.4)	0.823	(0.881)
	Girls	32	17.3	(17.43)	3.62	(2.2)	16.74	(18.9)	65.1	(53.3)	69.33	(64.4)		
**MEHHP**	**All**	56	26.73	(26.7)	6.4	(3.91)	24.1	(28.6)	80.5	(100.24)	129.85	(135.3)	0.387	(0.227)
	≥6~<12	22	28.9	(30.02)	14.31	(13.64)	27.03	(35.54)	74.8	(60.5)	95.7	(68.6)		
	≥12~≤18	34	25.4	(24.8)	6.4	(3.9)	21.92	(25.3)	80.5	(100.25)	129.85	(33.8)		
	Boys	24	29.03	(28.7)	10.61	(8.9)	30.9	(31.05)	95.7	(121.13)	129.85	(135.3)	0.602	(0.740)
	Girls	32	25.12	(25.3)	6.40	(3.91)	22.24	(26.8)	78.82	(61.9)	80.3	(78.03)		
**MEP**	**All**	56	27.5	(27.4)	13.1	(9.6)	28.2	(30.4)	53.5	(62.4)	62.1	(68.6)	0.491	(0.579)
	≥6~<12	22	30.2	(31.4)	18.7	(17.7)	28.7	(29.9)	53.5	(66.1)	62.1	(68.6)		
	≥12~≤18	34	25.8	(25.1)	6.3	(3.4)	26.9	(31.6)	51.2	(59.2)	54.9	(62.4)		
	Boys	24	23.5	(23.3)	13.1	(9.7)	25.5	(26.5)	53.5	(47.0)	54.9	(62.4)	0.012	(0.025)
	Girls	32	30.8	(31.1)	13.6	(9.6)	35.4	(34.2)	51.2	(66.1)	62.1	(68.6)		
**MBP**	**All**	56	36.4	(36.4)	12.8	(9.7)	42.9	(38.7)	70.3	(84.2)	72.3	(105.9)	0.502 (0.880)	
	≥6~<12	22	34.8	(36.2)	14.4	(13.1)	42.9	(41.6)	61.2	(71.3)	64.1	(72.0)		
	≥12~≤18	34	37.5	(36.5)	12.8	(9.8)	45.0	(36.9)	71.9	(98.1)	72.3	(105.9)		
	Boys	24	37.2	(36.8)	14.1	(9.8)	47.3	(39.2)	71.9	(98.1)	72.3	(105.9)	0.6429 (0.868)	
	Girls	32	35.9	(36.2)	12.8	(13.5)	38.5	(38.7)	69.7	(78.2)	70.3	(84.0)		
**MMP**	**All**	56	15.9	(15.8)	4.3	(4.3)	17.4	(15.8)	42.8	(48.2)	43.4	(53.8)	0.356 (0.737)	
	≥6~<12	22	14.5	(15.1)	5.2	(4.5)	15.2	(15.7)	34.2	(40.3)	40.0	(41.6)		
	≥12~≤18	34	16.8	(16.4)	4.3	(4.3)	19.3	(16.0)	43.3	(51.5)	43.4	(53.8)		
	Boys	24	15.8	(15.6)	4.3	(3.8)	19.1	(16.7)	40.0	(42.6)	42.7	(53.8)	0.8946 (0.973)	
	Girls	32	15.9	(16.1)	4.9	(4.5)	15.1	(15.2)	43.3	(48.2)	43.4	(51.5)		
**MBzP**	**All**	56	2.0	(2.0)	<LOQ		2.2	(2.3)	5.0	(5.4)	5.1	(6.2)	0.063 (0.043)	
	≥6~<12	22	2.4	(2.5)	0.7 (0.6)		3.4	(3.1)	5.0	(6.0)	5.1	(6.2)		
	≥12~≤18	34	1.8	(1.7)	<LOQ		2.1	(2.0)	4.4	(5.2)	5.1	(5.4)		
	Boys	24	1.8	(1.7)	0.7 (0.6)		2.0	(2.0)	4.5	(4.1)	5.1	(4.6)	0.0354 (0.043)	
	Girls	32	2.2	(2.2)	<LOQ		3.3	(2.8)	5.0	(6.0)	5.1	(6.2)		

Creatinine adjusted level of each phthalate metabolite was shown in the parentheses (μg/g); GM: geometric mean; LOQ: limit of quantification. LOQ for MzBP is 0.25 μg/L. ^a^ Mann-Whitney U test; *p*-value for adjusted level of each phthalate metabolite was shown in the parentheses.

**Table 4:** Correlations between urinary phthalate metabolite concentrations in (µg/g creatinine) among the Iranian children and adolescents population (*n* = 56).

Phthalate Metabolites	MBP	MEP	MMP	MEHP	MEOHP	MEHHP
MBzP	0.263 *	0.540 *	0.025	−0.003	0.127	0.021
MBP		0.277 *	0.625 *	−0.063	−0.000	0.043
MEP			0.006	0.187	0.163	0.119
MMP				0.026	−0.024	0.007
MEHP					0.731 *	0.798 *
MEOHP						0.896 *

* Correlation is significant at 0.05 levels (*p* < 0.05).

**Table 5:** Daily phthalate intakes (μg/kg body weight/day) estimated with the creatinine-based calculation model among the Iranian children and adolescents population (*n* = 56).

Phthalate Metabolites	Group	*n*	GM	5th	50th	95th	Max
DEHP	All	56	3.47	0.58	3.41	15.03	17.85
	≥6~<12	22	3.67	0.63	4.08	16.57	17.85
	≥12~≤18	34	3.34	0.49	3.39	15.16	16.06
	Boys	24	3.66	0.66	3.78	17.40	17.85
	Girls	32	3.33	0.47	3.39	11.25	11.74
DEP	All	56	0.78	0.23	0.88	1.85	2.56
	≥6~<12	22	0.84	0.40	0.86	2.45	2.56
	≥12~≤18	34	0.74	0.09	0.89	1.86	1.93
	Boys	24	0.64	0.20	0.66	1.78	1.93
	Girls	32	0.90	0.20	0.97	2.11	2.56
DBP	All	56	0.95	0.23	1. 1	2.47	3.10
	≥6~<12	22	0.87	0.20	1.11	2.10	2.16
	≥12~≤18	34	0.97	0.23	1.00	2.73	3.09
	Boys	24	0.91	0.19	0.97	2.97	3.09
	Girls	32	0.94	0.28	1.11	2.24	1.11
DMP	All	56	0.42	0.11	0.77	1.40	0.23
	≥6~<12	22	0.38	0.08	0.38	1.23	1.24
	≥12~≤18	34	0.45	0.11	0.44	1.86	1.5
	Boys	24	0.40	0.08	0.40	1.40	1.41
	Girls	32	0.44	0.12	0.42	1.44	1.54
BBP	All	56	0.06	0.008	0.06	0.16	0.23
	≥6~<12	22	0.07	0.09	0.07	0.22	0.23
	≥12~≤18	34	0.05	0.007	0.06	0.162	0.17
	Boys	24	0.05	0.012	0.06	0.11	0.112
	Girls	32	0.07	0.007	0.08	0.19	0.23

**Table 6:** Hazard quotients (HQ) and Hazard Index (HI) based on TDI (EFSA) and RfD-AA for Iranian children and adolescents.

Phthalates	Group	HQ TDI					HQ RfD-AA				
		Min	Median	95_p_	Max	*n* > 1	Min	Median	95_p_	Max	*n* > 1
DBP	**All**	0.02	0.11	0.25	0.31	0	0.001	0.01	0.02	0.03	0
	≥6~<12	0.02	0.11	0.21	0.22	0	0.002	0.01	0.02	0.02	0
	≥12~≤18	0.02	0.10	0.27	0.31	0	0.002	0.01	0.02	0.02	0
	Boys	0.02	0.10	0.30	0.31	0	0.002	0.01	0.03	0.03	0
	Girls	0.03	0.11	0.22	0.11	0	0.003	0.01	0.022	0.01	
BBP	**All**	0.00002	0.0001	0.0003	0.0005	0	0.00002	0.0002	0.0004	0.0007	0
	≥6~<12	0.0002	0.0001	0.0004	0.0005	0	0.00002	0.0002	0.0006	0.0007	0
	≥12~≤18	0.00001	0.0001	0.0003	0.0003	0	0.00002	0.0002	0.0005	0.0005	0
	Boys	0.00002	0.0001	0.0002	0.0002	0	0.00004	0.0002	0.0003	0.0003	0
	Girls	0.00001	0.0002	0.0004	0.0005	0	0.00002	0.0002	0.0006	0.0007	0
DEHP	**All**	0.01	0.10	0.31	0.40	0	0.02	0.11	0.50	0.60	0
	≥6~<12	0.01	0.10	0.33	0.36	0	0.02	0.14	0.55	0.60	0
	≥12~≤18	0.01	0.11	0.30	0.32	0	0.016	0.11	0.51	0.54	0
	Boys	0.01	0.10	0.35	0.40	0	0.02	0.13	0.58	0.60	0
	Girls	0.01	0.11	0.22	0.23	0	0.015	0.11	0.37	0.40	0
**HI**	**All**	0.03	0.20	0.56	0.70	0	0.02	0.13	0.52	0.62	0
	≥6~<12	0.03	0.20	0.54	0.60	0	0.03	0.14	0.57	0.62	0
	≥12~≤18	0.03	0.20	0.57	0.63	0	0.20	0.12	0.50	0.55	0
	Boys	0.03	0.20	0.65	0.70	0	0.03	0.13	0.60	0.62	0
	Girls	0.04	0.20	0.44	0.35	0	0.02	0.12	0.40	0.40	0

*n*: number of participants.
